# Selective REM Sleep Deprivation Improves Expectation-Related Placebo Analgesia

**DOI:** 10.1371/journal.pone.0144992

**Published:** 2015-12-17

**Authors:** Florian Chouchou, Jean-Marc Chauny, Pierre Rainville, Gilles J. Lavigne

**Affiliations:** 1 Faculties of Dental Medicine and Medicine, Université de Montréal, Montreal, Quebec, Canada; 2 Centre for Advanced Research in Sleep Medicine, Hôpital du Sacré-Coeur de Montréal, Montreal, Quebec, Canada; 3 Emergency Department, Hôpital du Sacré-Cœur de Montréal, Montreal, Quebec, Canada; St. Joseph's Hospital and Medical Center, UNITED STATES

## Abstract

The placebo effect is a neurobiological and psychophysiological process known to influence perceived pain relief. Optimization of placebo analgesia may contribute to the clinical efficacy and effectiveness of medication for acute and chronic pain management. We know that the placebo effect operates through two main mechanisms, expectations and learning, which is also influenced by sleep. Moreover, a recent study suggested that rapid eye movement (REM) sleep is associated with modulation of expectation-mediated placebo analgesia. We examined placebo analgesia following pharmacological REM sleep deprivation and we tested the hypothesis that relief expectations and placebo analgesia would be improved by experimental REM sleep deprivation in healthy volunteers. Following an adaptive night in a sleep laboratory, 26 healthy volunteers underwent classical experimental placebo analgesic conditioning in the evening combined with pharmacological REM sleep deprivation (clonidine: 13 volunteers or inert control pill: 13 volunteers). Medication was administered in a double-blind manner at bedtime, and placebo analgesia was tested in the morning. Results revealed that 1) placebo analgesia improved with REM sleep deprivation; 2) pain relief expectations did not differ between REM sleep deprivation and control groups; and 3) REM sleep moderated the relationship between pain relief expectations and placebo analgesia. These results support the putative role of REM sleep in modulating placebo analgesia. The mechanisms involved in these improvements in placebo analgesia and pain relief following selective REM sleep deprivation should be further investigated.

## Introduction

The placebo effect is a neurobiological and psychophysiological process known to reduce pain perception. This analgesia effect is mediated through two main mechanisms, expectations and learning, which influence pain relief independently of the treatment effect itself [1]. This may be especially relevant in clinical acute and chronic pain management [2], where potential avenues to improve pain treatment efficacy include identifying factors that contribute to placebo analgesia [3].

Much evidence supports the attribution of the placebo effect to a wide range of neurobiological and psychophysiological processes [1,4,5,6], including learning from classical conditioning to social learning (i.e., vicarious learning) [7,8], expectancy [9], emotional regulation [6], and reward and motivational mechanisms [10]. For example, positive expectations of clinical benefits of treatment is suggested to reduce anxiety, increase motivation, and activate reward circuits in the brain, which may contribute in turn to reduce symptoms. Furthermore, learning—especially classical conditioning—may act on pain appraisal by reinforcing relief expectations [8,11]. According to the conditioning—expectancy relationship, experimental placebo tests could be designed as a series of extinction trials (i.e., conditioned stimulus presented without a decrease in nociceptive input). In this view, the maintenance of hypoalgesia reflects the preserved relief expectations preserved classical extinction processes [11].

Certain types of brain activity during sleep appear to play a role in some of the psychological and neurobiological processes involved in placebo effects [9,12–14]. Non-rapid eye movement (NREM) sleep, characterized by slow electroencephalographic rhythms such as delta waves, sleep spindles, and K-complexes, is suggested to be associated with declarative memory processes [9,13]. In contrast, rapid eye movement (REM) sleep is characterized by low-amplitude, high-frequency electroencephalographic rhythms, muscular hypotonia, and brain activity more similar to wakefulness. This sleep stage is associated with emotional, procedural, and conditioning memory processes, although the evidence is relatively scarce [12,14–17]. In our previous study, correlation analyses suggested that individuals with *less* REM sleep showed stronger expectation-mediated placebo analgesia in the following morning. REM sleep was therefore suggested to contribute to associative information reprocessing (e.g., following a conditioning procedure), which may have down-regulated the expectation-related placebo response [18]. These findings underscore that sleep-related processes may influence the association between pain relief expectations and placebo analgesia. Further studies are needed to confirm the involvement of REM sleep in placebo-induced subjects with pain relief expectations to determine causal relationships between REM sleep, placebo-induced expectations, and pain relief.

REM sleep deprivation (REMSD) is particularly relevant for examining the relationship between sleep and placebo analgesia. However, sleep disruptions (e.g., total and partial sleep restriction, awakenings during sleep) are known to alter pain sensitivity the next day [19]. A pharmacological REMSD model would be more suitable than the usual awakening from REM sleep method [20] for investigating pain perception and placebo analgesia. We therefore used a pharmacological REM sleep deprivation design using clonidine, an alpha-2 adrenergic receptor agonist known to suppress most of REM sleep in a majority of subjects, with no changes in sleep duration or sleep fragmentation [21–23].

In the present study, we used clonidine as a pharmacological tool to induce REM sleep deprivation. The objective was to experimentally challenge the role of REM sleep in the placebo response to analgesia suggestion. Clonidine is an alpha-2 adrenergic receptor agonist known to suppress most of REM sleep in animals [24] and humans [21–23]. Clonidine use in healthy volunteers does not interfere with sleep duration or trigger a rise in sleep fragmentation in relation to REM sleep deprivation.

The aim of this study was to determine the impact of experimental REMSD on expectation-related placebo analgesia. Based on previous studies, we hypothesized that expectation-related placebo analgesia would be improved by experimental REMSD in healthy volunteers.

## Materials and Methods

### Participants

Twenty-six healthy subjects (aged 23.4 ± 0.6 (standard error (SEM) years, range: 20–31, 12 women, 3 left-handed) were recruited through poster advertisements at various faculties and departments of the Université de Montréal. Inclusion criteria were a history free of chronic pain or neurological, psychiatric, or sleep disorders. No medication consumption was allowed during the study, except for contraceptive pills. Participants were asked to abstain from caffeine and alcohol for 24 hours before each recording night and testing day.

**Fig 1:**
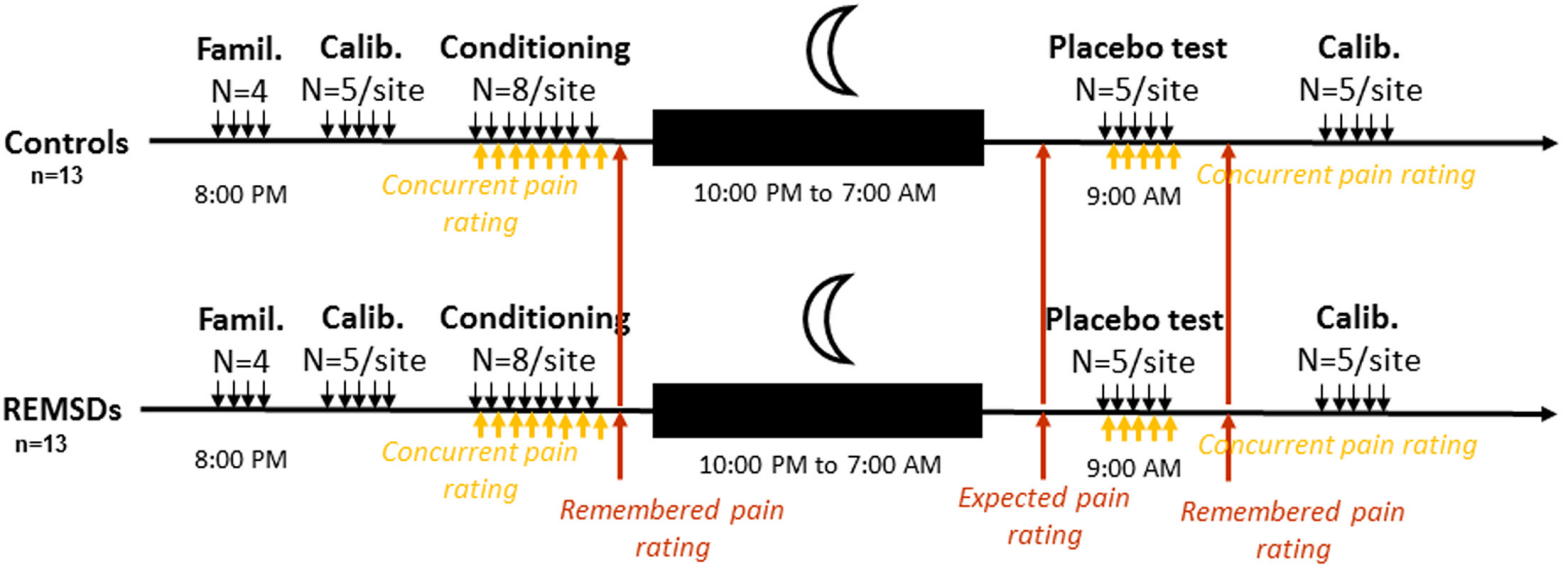
Time course of experimental events: Stimuli were administered in four separate experimental blocks: familiarization, calibration, conditioning, and testing. Treatment (clonidine for REMSDs (n = 13) or placebo for control pill group (n = 13)) was given immediately before going to bed. Famil: familiarization, Calib: calibration.

Before the study, subjects underwent a standard clinical examination under medical supervision to prevent hypotension associated with clonidine administration. Twelve-lead electrocardiograms were recorded and office blood pressure was measured using a mercury sphygmomanometer: 1) after lying for 15 minutes, 2) once immediately after assuming upright position, and 3) at two 5-minute intervals afterward. Subjects were excluded if they presented hypotensive sensitivity (systolic blood pressure < 105 mmHg, diastolic blood pressure < 55 mmHg) (5 exclusions) or cardiac anomaly (1 exclusion) (see Experimental design and Fig 1).

This study was approved by the ethics committee of Sacré-Cœur Hospital, and all subjects gave their informed consent according to institutional rules.

### Experimental design

The study was conducted at the Centre for the Study of Advanced Research for Sleep Medicine, Sacré-Cœur Hospital (Montreal, Quebec, Canada). After clinical examination, subjects underwent an initial nocturnal polysomnographic recording to control for the presence of sleep disorders such as sleep-disordered breathing, movement disorders (periodic limb movements, bruxism), and other sleep anomalies. One subject was excluded for sleep apnea syndrome, with an apnoea-hypopnoea index of > 15 events per hour. Subjects were invited for a second recording night one week later, and were administered either clonidine 0.3 mg per os or an inert control pill in a double-blind randomized sequence to create two groups: one experimental (REMSDs, n = 13) and one control (Controls, n = 13). The active clonidine pill and the control pill were identical in shape and color (blue-and-white capsule) and were presented to subjects in an envelope designated by a number according to the time of study inclusion. The procedure was identical for both groups and was administered by the same investigator (FC), who was blind to the experimental condition. Polysomnographic data were recorded on a computer and were not accessible to the experimenter to prevent breaking the blind of clonidine/control pill effect for the morning placebo analgesia tests.

Before bedtime, subjects underwent placebo conditioning (see Experimental procedure and Fig 1) before taking a pill. They were informed that “The pill you were given is either clonidine or a placebo, an inactive substance. Neither you nor I know which type of the pill you have been given. If you were given clonidine, your sleep will be affected without your noticing it, and with no harmful effects on you tomorrow. During the screening visit, before you signed the consent form, you were informed that clonidine may lower your blood pressure, and we will therefore monitor your blood pressure in the morning.”

Lights in the experimental sleep bedroom were turned off at 10:00 P.M., and subjects were woken up at 7:00 A.M. the next morning. Two hours after being woken, subjects underwent placebo testing blocks (see Experimental procedure and Fig 1). Before subjects left the laboratory, they were debriefed on the placebo conditioning experience.

### Experimental procedure

Our group previously used [18] a similar placebo analgesia experimental design consisting of four separate blocks of pain trials: familiarization, calibration, conditioning, and placebo testing, based on the stimulation protocol developed by Price and collaborators [25]. Familiarization, calibration, and conditioning were performed in the evening, with placebo testing and calibration in the morning. During the familiarization block, subjects underwent one trial each of thermal stimuli administered at 44, 45, 47, and 49°C on each arm to become gradually accustomed to the stimuli. In the calibration block, series of stimulations were delivered to each arm using a graphic method of limits to determine individually adjusted temperatures for each participant during the conditioning and experimental blocks. Two sites of cutaneous stimulation over each forearm were used. A 3 cm^2^ probe (NeuroSensory Analyser TSA-II; Medoc Ltd.) was used to induce contact heat pain (44–49°C) from a baseline temperature of 32°C.

At the beginning of the conditioning and placebo blocks, the same inert cream was applied to control and treatment sites on each arm. For the treated site (placebo site), the cream was described to the subject as a topical analgesic, and for the control site it was described as an inert cream to control for nonspecific effects of the vehicle compound. The placebo condition was assigned to the dominant arm in half the subjects of each group. In both the conditioning and placebo test blocks, successive phasic stimuli were delivered at increasing temperature from the 32°C baseline, at an incremental rate of 4°C/s and maintained for 7 sec at target intensity. Each stimulus onset was preceded by a 5 sec auditory countdown, and successive stimulus onsets were separated by 60 sec intervals to minimize the risk of local sensitization. During the conditioning block, a sequence of 8 stimuli was delivered to both the control and placebo site. Whereas the control site was stimulated at a moderate pain level (60/100 on the pain intensity scale) determined individually based on the first calibration block, the temperature of the stimuli applied to the placebo site was surreptitiously decreased by 2°C below that of the control site. The purpose was to provide an unambiguous analgesia experience. Immediately before the conditioning phase, the cream applied to the placebo site was taken from a container with a pharmacy label and applied using a cotton bud. This cream was described to the participant as a topical analgesic. The cream applied to the control site was applied in the same manner but was taken from an unlabeled container. It was described as an inert cream to control for nonspecific effects of the vehicle compound of the analgesic cream [18]. Familiarization and calibration blocks were performed at 7:00 P.M., and conditioning began at 8:00 P.M. In the morning placebo testing block, subjects received 5 thermal stimuli on each arm at the same predetermined moderate pain stimulation level. Placebo analgesia was assessed 2 h after waking (9:00 A.M.). At the conclusion of these pain trials, another calibration block was administered to all subjects to assess potential circadian phase-related variations in pain sensitivity.

### Pain ratings

Subjective assessments of pain intensity and unpleasantness were obtained using a 15 cm mechanical visual analog scale (VAS) [26]. VAS pain intensity ratings were rated by responding to the question “How intense was the pain?” on a scale ranging from “no pain sensation” to “most intense pain imaginable.” Similarly, VAS pain unpleasantness was rated by responding to the question “How much did the stimulation bother you?” on a scale ranging from “not at iall unpleasant” to “most unpleasant pain imaginable.” These two scales were also used to rate expected and remembered pain.

*Expected pain*. Expected pain intensity and unpleasantness were prospectively obtained at the beginning of the conditioning and placebo testing blocks. Subjects were asked, “What do you expect the pain intensity/unpleasantness to be without/with the analgesic cream?”

*Concurrent pain*. In the conditioning and testing blocks, subjects were asked to rate the intensity and unpleasantness of the pain felt immediately after each stimulation.

*Remembered pain*. Approximately 2 min after the end of the placebo testing block, subjects were asked to retrospectively rate the overall pain felt at the control and placebo sites: “Retrospectively, what was the overall pain intensity/unpleasantness you felt without/with the analgesic cream?”

### Sleep study

Standard in-hospital polysomnographic recordings were performed using a polygraphic device (Harmony^®^, formerly Stellate System, Canada, Natus, USA) and scored according to AASM recommendations [27]. A total of 12 electroencephalogram leads (Fp_1_-M_2_; Fp_2_-M_1_; F_3_-M_2_; F_4_-M_1_; Fz-M_2_; C_3_-M_2_; C_4_-M_1_; Cz-M_1_; P_3_-M_2_; P_4_-M_1_; O_1_-M_2_; O_2_-M_1_), 5 electromyogram channels (3 for chin and 1 for each leg), 2 electro-oculogram channels (E_1_-M_2_; E_2_-M_2_), 3 electrocardiogram channels (D1, D2, D3), 2 piezoelectric belts for chest and abdominal efforts, 1 nasal cannula, and 1 oxygen saturation channel were used. Electroencephalogram, electro-oculogram, electromyogram, and electrocardiogram data were collected at a sampling rate of 500 Hz and respiration was monitored by nasal cannula at 200 Hz, with two piezoelectric belts (10 Hz) for chest and abdominal effort and one oxygen saturation channel (1 Hz). A ground was placed on the mid-forehead. Skin impedance was kept below 5 kiloOhms for all electrodes and recorded signals were filtered at 70 Hz (low pass) with 1-sec time constant and digitized at a sampling rate of 500 Hz. Electrophysiological and respiratory data were recorded continuously from 10:00 P.M. to 07:00 A.M. and stored for later analysis.

Sleep stages were visually scored by a trained technician blind to the hypothesis, and were based on 30 sec epochs, as follows: stage 1, stage 2, slow wave sleep, and REM sleep. Micro-arousals were defined as bursts of wakefulness cortical activity lasting from 3 to 15 sec, with bursts lasting more than 15 sec considered as arousals. Calculated sleep parameters also included total sleep time, wake time after sleep onset, sleep efficiency (total sleep time/total recording time*100), and duration of sleep stage 1, sleep stage 2, slow wave sleep, and REM sleep. We further assessed subjective sleep quality on a VAS (0–10) at awakening.

### Psychomotor vigilance task and questionnaires

To control for interactions between fluctuations in vigilance, subjective sleepiness, and treatment (REMSDs vs. Controls), the Karolinska [28] and Epworth [29] Sleepiness Scale and a 10 min psychomotor vigilance task [30] were administered to all subjects in the evening and morning before pain trial blocks. Overall questionnaire scores and median and mean of reactions times were considered.

### Blood pressure monitoring

Blood pressure (BP) was systematically measured with a mercury sphygmomanometer on the non-dominant arm in lying position after a 15-minute rest. BP was measured in the evening before pain trial blocks and in the morning after placebo testing blocks. BP was measured in the evening before pain trial blocks and twice in the morning: at awakening and immediately after placebo testing blocks. Systolic (SAP) and (DAP) diastolic BP were calculated evenings and mornings (1 and 2).

### Statistical analysis

Data were presented as mean ± standard error (SEM) and analyzed using Statview^®^ (SAS Institute, Inc. Cary, NC, USA) and SPSS^®^ (IBM Inc., NY, USA). All statistical tests were performed at p < 0.05. To assess the reliability of our REMSD model, sleep parameters were compared using two-sided Kruskal—Wallis tests or two-sided ANOVAs when appropriate. To assess score differences in pain calibration tests, questionnaires, BP, and psychomotor vigilance tasks according to both time (evening vs. morning) and groups, we used repeated ANOVAs with group as the between factor.

Expected, concurrent, and remembered pain intensity and unpleasantness ratings obtained in the conditioning and placebo test blocks were compared using two-sided ANOVAs (placebo and control sites) with group as the between factor (REMSDs and Controls). The placebo effects for expected, concurrent, and remembered pain intensity and unpleasantness ratings obtained in the conditioning and placebo test blocks were compared using two-sided ANOVAs with group as the between factor.

To assess the moderating effect of REM (M) on the relationship between expectations (X) and placebo analgesia (Y), we tested the simplest model described in Hayes (2013; Model 1) [31] using the Process macro (v. 2.13) in SPSS (v. 22) (http://www.processmacro.org/). We defined X as the difference in expected pain intensity (Exp Analg Int) or unpleasantness (Exp Analg Unp) between the experimental and control arm (i.e., magnitude of analgesic expectations). Two models were tested for pain intensity, with the outcome variable Y defined as the placebo analgesic effect (experimental vs. control arm) on concurrent (Conc Analg Int) or remembered (Remb Analg Int) pain intensity. Two similar models were also tested using unpleasantness expectations and the outcome variable Y defined as the placebo analgesic effect on concurrent (Conc Analg Unp) or remembered unpleasantness (Remb Analg Unp). In a first set of analyses A, REM sleep duration was treated as a continuous moderator variable M; in a second step B, the REMSD group was treated as a categorical moderator variable M affecting the relationship between X and Y; and in a third step C, REM sleep duration was treated as a continuous moderator variable M, and the model was tested in the control group only to determine whether the moderating effect of REM duration remained relevant in the absence of sleep disruption.

## Results

### Calibration blocks

After familiarization blocks, calibration tests for stimulation intensity were performed to induce moderate pain (60% VAS pain intensity) for the remainder of the protocol (see Fig 1). Because the experimental design involved pain stimuli applied morning and evening, morning calibration blocks were also performed to assess potential circadian phase-related variations in pain. Repeated ANOVAs showed that temperatures required to induce moderate pain were similar (F(1,25) = 0.09, p = 0.772, interaction: F(1,25) = 0.06, p = 0.808) between REMSDs and Controls and between evening and morning temperature (F(1,25) = 0.96, p = 0.336) (evening, REMSDs: 47.0 ± 0.4°C, Controls: 46.8 ± 0.3°C; morning, REMSDs: 47.2 ± 0.5°C, Controls: 47.0 ± 0.3°C).

### Placebo conditioning before sleep and before medication intake

Individual stimulus intensities during placebo conditioning were adjusted based on the first calibration block to generate moderate pain at the control site (60% VAS pain intensity) and lower pain at the placebo site (-2°C). Overall mean temperatures for the conditioning block at placebo and control testing sites were 47.0 ± 0.3 and 45.0 ± 0.3°C, respectively, for REMSDs and 46.8 ± 0.4 and 44.8 ± 0.4°C, respectively, for Controls.

Subjects in both groups (clonidine or control pill) rated comparable concurrent pain intensity during conditioning (F(1,25) = 1.96, p = 0.175) and comparable remembered pain during conditioning (F(1,25) = 1.78, p = 0.195). Similarly, concurrent pain unpleasantness ratings were comparable during conditioning (F(1,25) = 3.35, p = 0.080), as were remembered pain unpleasantness ratings during conditioning (F(1,25) = 3.79, p = 0.064). As expected, both groups rated lower concurrent (site effect: F(1,25) = 78.9, p < 0.001, interaction: F(1,25) = 0.13, p = 0.720) and remembered (site effect: F(1,25) = 74.9, p < 0.001, interaction: F(1,25) = 0.01, p = 0.939) pain intensity at the placebo versus the control site. Similar ratings were reported for concurrent (site effect: F(1,25) = 75.08, p < 0.001, interaction: F(1,25) = 0.13, p = 0.727) and remembered (site effect: F(1,25) = 60.69, p < 0.001, interaction: F(1,25) <0.01, p = 0.992) pain unpleasantness at the placebo versus control site.

Both groups also reported comparable analgesia (control placebo sites) induced by suggestion and placebo cream during conditioning in concurrent (F(1,25) = 0.14, p = 0.710) and remembered (F(1,25) < 0.01, p = 0.939) pain intensity ratings. Similarly, both groups reported comparable analgesia (control vs. placebo sites) in concurrent (F(1,25) = 0.13, p = 0.727) and remembered (F(1,25) < 0.01, p = 0.992) pain unpleasantness ratings.

These results indicate that, before taking the medication and sleeping, conditioning induced comparable pain relief across the two groups, and all subjects in both groups had similar initial analgesia expectations with the proposed treatment in the evening.

### Sleep deprivation

To control for the effect of clonidine on sleep, several sleep parameters were assessed, as reported in Table 1. To summarize, REM sleep duration was shorter (p < 0.001) in REMSDs (Controls: 16.9 ± 0.7%; REMSDs: 0.9 ± 0.4%, 7/13 subjects with no REM sleep). Conversely, REMSDs showed longer sleep stage 2 compared to Controls (Controls: 48.6 ± 1.1%; REMSDs: 61.8 ± 2.5%, p < 0.001). However, total sleep and slow wave sleep durations (p = 0.238 and p = 0.149, respectively) did not differ between groups, nor did sleep fragmentation (arousals (p = 0.664); micro-arousals (p = 0.407)). Overall, all subjects in both groups reported comparably good sleep quality in the morning assessed by VAS (REMSDs: 6.17 ± 0.53 VAS units (/10); Controls: 6.65 ± 0.39 VAS units (/10)). As expected, these results indicate that clonidine medication strongly decreases or suppresses REM sleep, with no alterations in sleep continuity, architecture, or quality.

**Table 1:** Polysomnographic parameters for both groups (mean ± standard error of the mean). Two-sided Kruskal—Wallis tests or two-sided ANOVAs were used according to the normal distribution of the variable.

	REMSDs		Controls		
	Mean	SEM	Mean	SEM	p
Sleep efficiency (%)	92.94	1.13	92.26	1.39	0.898
Sleep latency (min)	10.00	2.6	20.96	5.60	0.237
Total sleep time (min)	495.5	6.9	480.9	9.7	0.238
Stage 1 (%)	6.5	0.9	6.7	0.5	0.698
Stage 2 (%)	61.8	2.5	48.6	1.1	**< 0.001**
Stage 3 (%)	29.4	1.5	21.0	1.5	0.149
REM sleep (%)	0.9	0.4	16.9	0.7	**< 0.001**
Arousal (n)	33.08	6.46	39.73	7.12	0.644
Micro-arousal index (n/h)	9.25	0.79	10.81	1.66	0.407
Sleep quality (/10)	6.17	0.53	6.65	0.39	0.463

### Expected placebo analgesia

**Fig 2:**
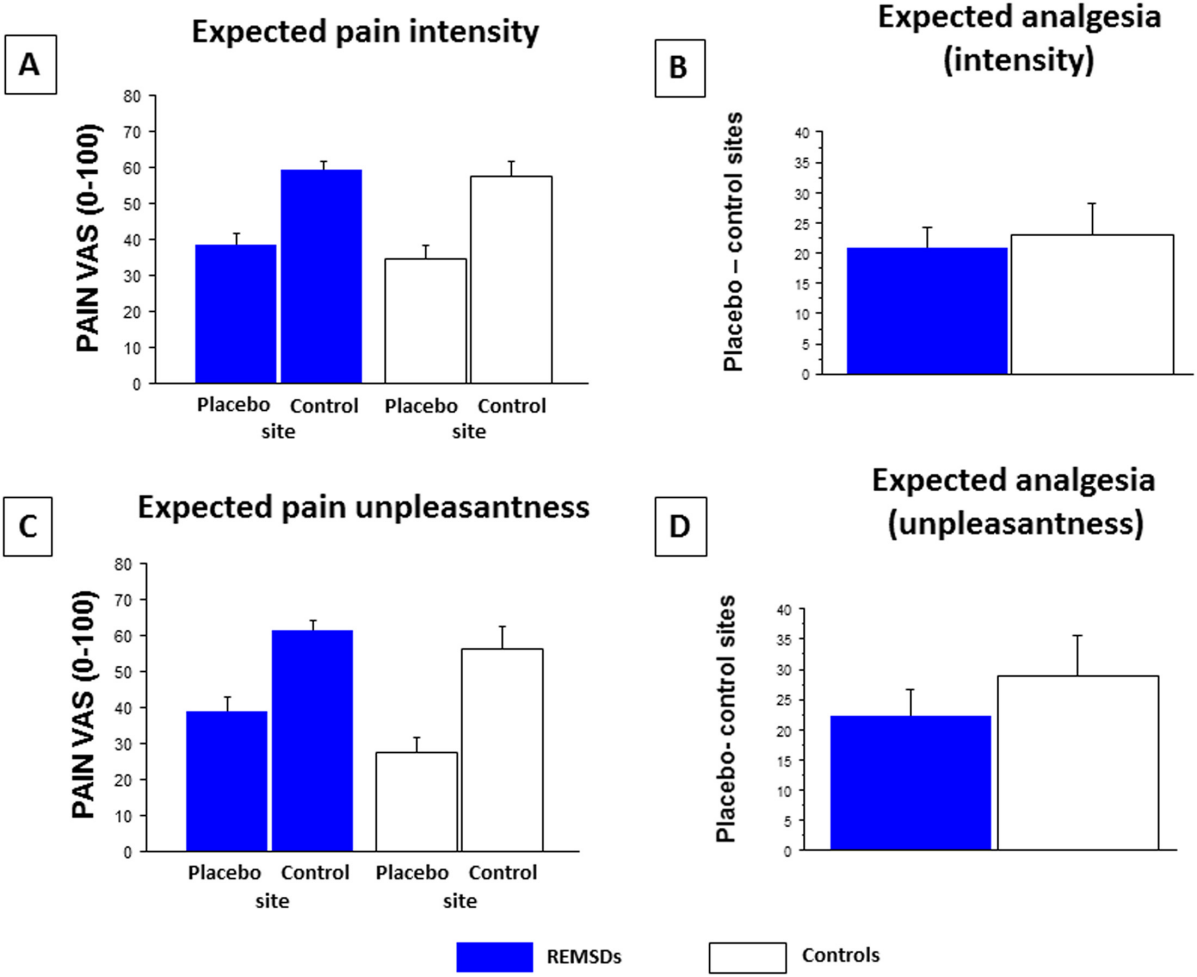
**Expected pain A) intensity and B) unpleasantness measured in the morning at control and placebo test sites before placebo testing block for REMSDs and Controls.** Statistical analyses revealed no group effect for expected pain intensity or unpleasantness (REMSDs vs. Controls, p = 0.501 and 0.126), but a stimulation site effect was found (control vs. placebo sites: p < 0.001 and p < 0.001) with no interaction (p = 0.761 and 0.415). **Analysis of expected pain intensity (C) and unpleasantness (D) between placebo and control test sites** revealed no significant between-group difference (p = 0.927 and p = 0.276) (mean ± standard error of the mean). Subjective assessments of pain intensity and unpleasantness were obtained by visual analog scale (VAS, 0–100).

**Fig 3:**
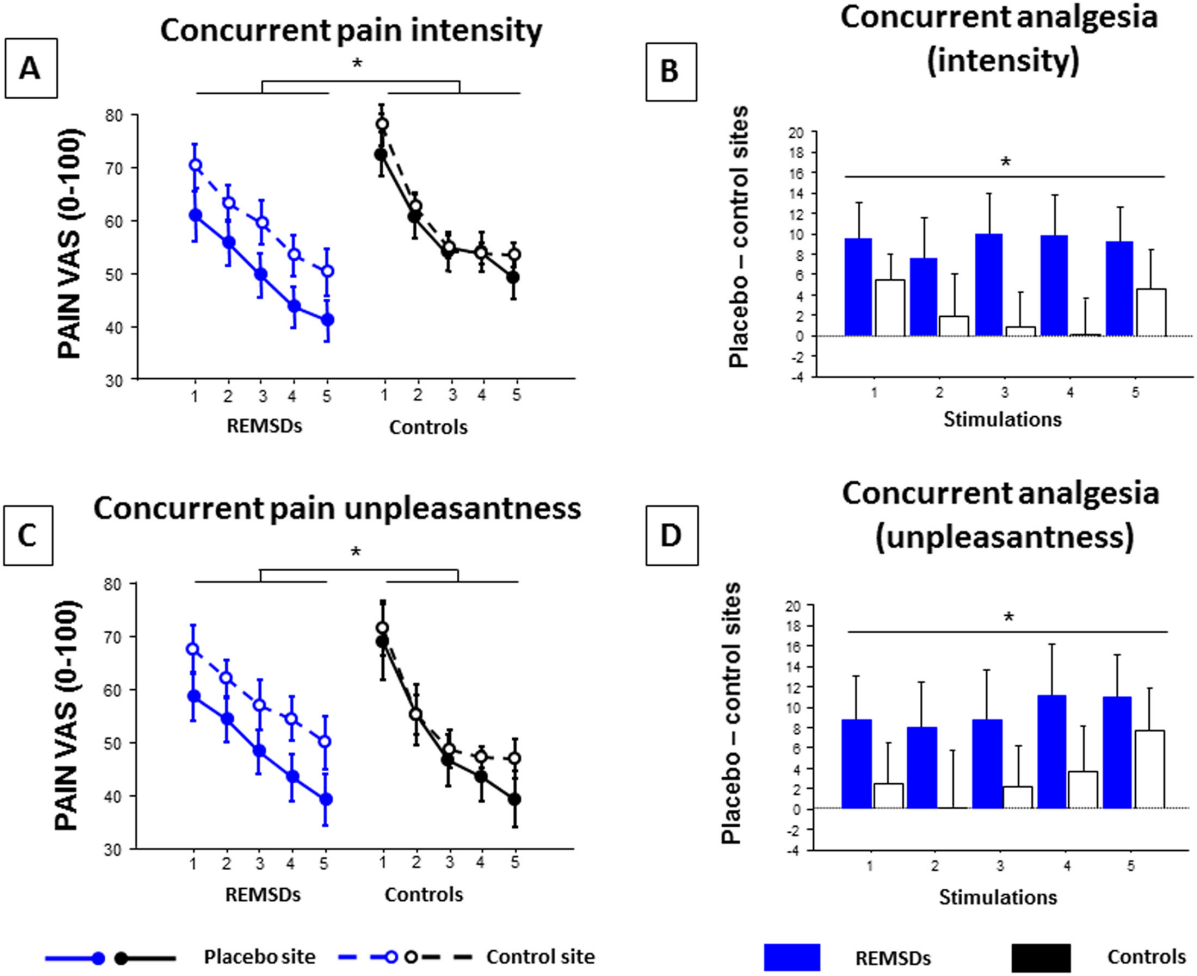
**Concurrent pain A) intensity and B) unpleasantness measured in the morning at control and placebo sites for REMSDs and Controls.** Statistical analyses revealed a group effect for concurrent pain intensity and unpleasantness (REMSDs vs. Controls, p = 0.028 and 0.030), a stimulation site effect (control vs. placebo sites: p < 0.001 and p < 0.001), and an interaction (*: p = 0.006 and 0.030). **Analysis of concurrent pain intensity (C) and unpleasantness (D) between placebo and control sites** revealed a significant between-group difference (p = 0.006 and p = 0.030) (mean ± standard error of the mean). 1 to 5 depict stimulations 1 to 5. Subjective assessments of pain intensity and unpleasantness were obtained by visual analog scale (VAS, 0–100).

**Fig 4:**
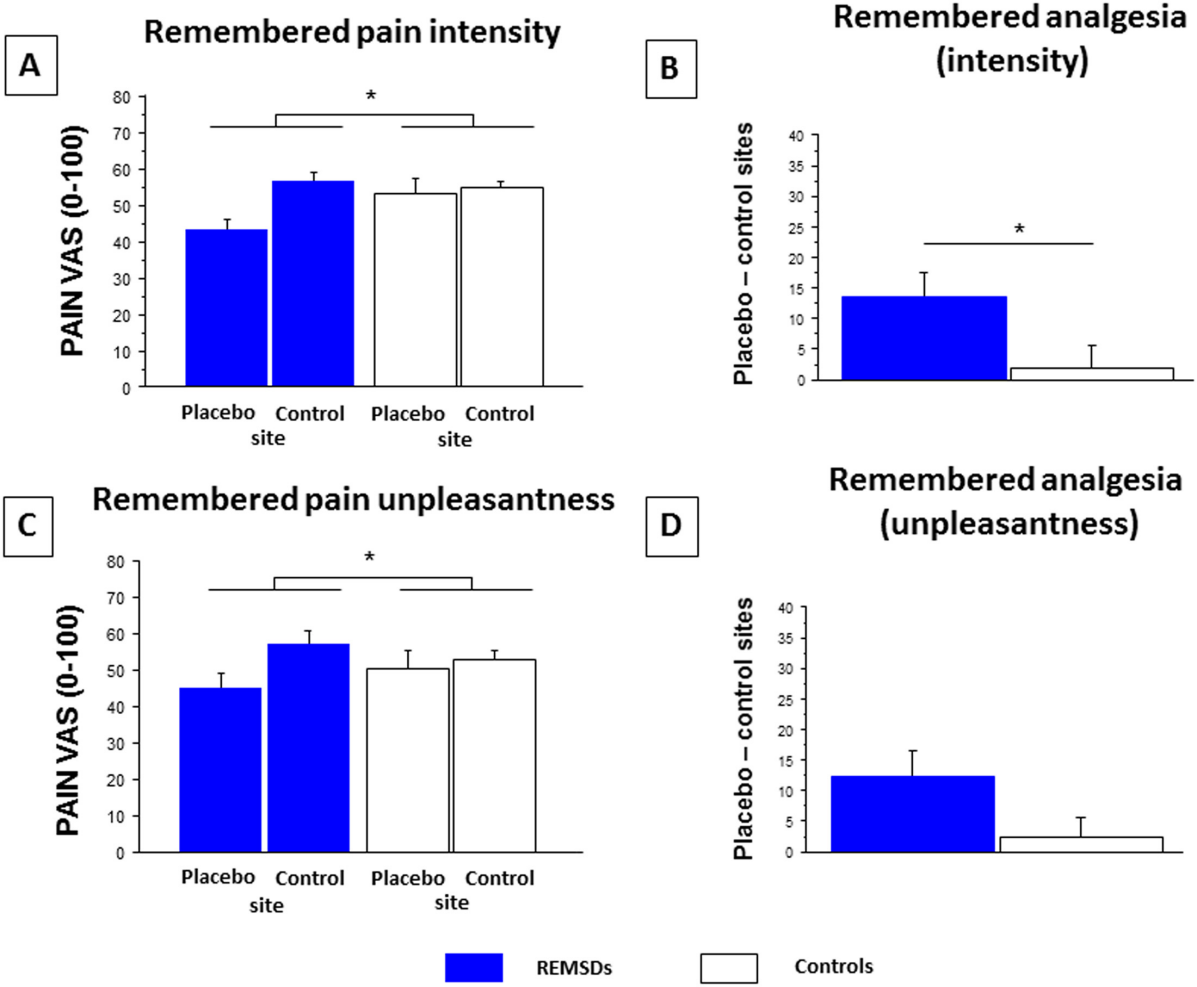
**Remembered pain A) intensity and B) unpleasantness measured in the morning at control and placebo sites for REMSDs and Controls.** Statistical analyses revealed a group effect for concurrent pain intensity and unpleasantness (REMSDs vs. Controls, p = 0.028 and 0.030), a stimulation site effect (control vs. placebo test sites: p < 0.001 and p < 0.001), and an interaction (p = 0.006 and 0.030). **Analysis of remembered pain intensity (C) and unpleasantness (D) between placebo and control sites** revealed a significant between-group difference (p = 0.002) for pain intensity, but not for unpleasantness (p = 0.070) (mean ± standard error of the mean). Subjective assessments of pain intensity and unpleasantness were obtained using visual analog scale (VAS, 0–100).

Relief expectations, pain perceptions, and placebo analgesia were assessed in the morning for each group (clonidine or control pill). Expected, concurrent, and remembered pain intensity and unpleasantness ratings for control and placebo sites are summarized in Figs 2–4, respectively. Results show that both groups expected similar moderate pain intensity at the control site and low pain intensity at the placebo site (F(1,25) = 0.47, p = 0.501; site effect: F(1,25) = 48.89, p < 0.001; interaction: F(1,25) = 0.94, p = 0.761). Thus, both groups expected a similar degree of analgesia (F(1,25) = 0.94, p = 0.761). Similar results were found for expected pain unpleasantness ratings: both groups expected similar moderate pain unpleasantness at the control site and low pain unpleasantness at the placebo site (group effect: F(1,25) = 2.52, p = 0.126; site effect: F(1,25) = 40.13, p < 0.001; interaction: F(1,25) = 0.68, p = 0.415). Thus, similar unpleasantness analgesia was expected across both groups (F(1,25) = 0.94, p = 0.415).

Conversely, subjects reported lower concurrent pain intensity at the placebo site following REMSD compared to Controls (group effect: F(1,25) = 3.51, p = 0.06; site effect: F(1,25) = 25.83, p < 0.001; interaction: F(1,25) = 8.37, p = 0.005). Thus, placebo analgesia was higher following REMSD compared to Controls (F(1,25) = 8.37, p = 0.005). Similar results were found for concurrent pain unpleasantness ratings (group effect: F(1,25) = 0.18, p = 0.676; site effect: F(1,25) = 20.02, p < 0.001; interaction: F(1,25) = 5.04, p = 0.027), with higher placebo analgesia following REMSD (F(1,25) = 5.04, p = 0.003).

Subjects also reported lower remembered pain intensity at the placebo site following REMSD (group effect: F(1,25) = 1.68, p = 0.207; site effect: F(1,25) = 8.16, p = 0.009; interaction: F(1,25) = 4.59, p = 0.046) and higher placebo analgesia (F(1,25) = 4.59, p = 0.046). Remembered pain unpleasantness ratings also tended toward significance (group effect: F(1,25) = 0.01, p = 0.907; site effect: F(1,25) = 8.00, p = 0.009; interaction: F(1,25) = 3.56, p = 0.072), along with remembered analgesia (p = 0.072).

These results demonstrate a significant improvement in the placebo effect following REMSD and conditioning, with no change in pain relief expectations.

### REM sleep as a moderator of the relationship between expectations and analgesia

To assess the effect of REMSD on the relationship between expectations and placebo analgesia, we used the simplest Hayes (2013) model [31], with the moderator variable defined as REM sleep duration (Table 2A and 2C) or (REMSDs and Controls; Table 2B).

**Table 2:** Results of the moderation analysis testing the effect of REM (M) on the relationship between expected (X) and placebo (Y) analgesia.

Outcome Y^a^	Predictor X^b^	Moderator M	Sample n =	Effect of X p =	Interact. XM p =	Conditional effect of moderator on X to Y^c^	
***A*. *Effects of expectations on placebo analgesia are moderated by REM duration***							
*Conc Analg Int*	*Exp Analg Int*	*REM duration*	26	**0.008**	**0.02**	**Low REM**	**p = 0.008**
						**Moderate REM**	**p = 0.005**
						High REM	p = 0.22
*Remb Analg Int*	*Exp Analg Int*	*REM duration*	26	**0.0001**	**0.01**	**Low REM**	**p = 0.0001**
						**Moderate REM**	**p < 0.0001**
						High REM	p = 0.05
*Conc Analg Unp*	*Exp Analg Unp*	*REM duration*	26	**0.0001**	**0.003**	**Low REM**	**p = 0.001**
						**Moderate REM**	**p = 0.002**
						High REM	p = 0.36
*Remb Analg Unp*	*Exp Analg Unp*	*REM duration*	26	**<0.0001**	**0.0002**	**Low REM**	**p < 0.0001**
						**Moderate REM**	**p < 0.0001**
						High REM	p = 0.13
***B*. *Effects of expectations on placebo analgesia are moderated by REMSD Group***							
*Conc Analg Int*	*Exp Analg Int*	*REMSDs*	26	0.34	*0*.*054*	**REMSDs**	**p = 0.004**
						Controls	p = 0.18
*Remb Analg Int*	*Exp Analg Int*	*REMSDs*	26	0.51	*0*.*057*	**REMSDs**	**p = 0.001**
						**Controls**	**p = 0.04**
*Conc Analg Unp*	*Exp Analg Unp*	*REMSDs*	26	0.10	**0.008**	***REMSDs***	**p = 0.0005**
						Controls	p = 0.32
*Remb Analg Unp*	*Exp Analg Unp*	*REMSDs*	26	*0*.*06*	**0.002**	***REMSDs***	**p = 0.0001**
						Controls	p = 0.13
***C*. *Effects of expectations on placebo analgesia are moderated by REM duration in the control group***							
*Conc Analg Int*	*Exp Analg Int*	*REM duration*	13	0.10	0.13	**Low REM**	**p = 0.051**
						Moderate REM	p = 0.14
						High REM	p = 0.64
*Remb Analg Int*	*Exp Analg Int*	*REM duration*	13	**0.03**	0.052	**Low REM**	**p = 0.009**
						**Moderate REM**	**p = 0.03**
						High REM	p = 0.69
*Conc Analg Unp*	*Exp Analg Unp*	*REM duration*	13	**0.03**	**0.04**	**Low REM**	**p = 0.02**
						Moderate REM	p = 0.13
						High REM	p = 0.29
*Remb Analg Unp*	*Exp Analg Unp*	*REM duration*	13	**0.04**	0.06	**Low REM**	**p = 0.02**
						Moderate REM	p = 0.10
						High REM	p = 0.44

^a^. Conc Analg Int: difference in concurrent pain intensity between experimental and control arm; Remb Analg Int: difference in remembered pain intensity between experimental and control arm; Conc Analg Unp: difference in concurrent pain unpleasantness between experimental and control arm; Remb Analg Unp: difference in remembered pain unpleasantness between experimental and control arm.

^b^. Exp Analg Int: difference in expected pain intensity between experimental and control arm; Exp Analg Unp: difference in expected pain unpleasantness between experimental and control arm.

^c^. Conditional effects are reported at the mean—1SD REM duration (Low REM), mean REM duration (Moderate REM), and mean + 1 SD REM duration (High REM).

We first examined the moderating effect of REM sleep duration as a parametric moderator across all subjects. Results showed that REM sleep duration significantly affected the relationship between expected and both concurrent and remembered unpleasantness (interaction term: p ≤ 0.003, Table 2A). A similar moderating effect was also found on pain intensity (p ≤ 0.02, Table 2A). Subsequent conditional analysis confirmed that expectations predicted placebo analgesia for concurrent and remembered pain intensity and unpleasantness for low and moderate REM duration (p ≤ 0.005), but not for high REM duration (p ≥ 0.05). However, a closer examination of REM duration across all participants showed that this variable was not normally distributed due to the clonidine manipulation (i.e., 7 subjects in the REMSD group had REM duration = 0). We therefore further tested REM as a categorical moderator, and results confirmed that the REMSD group significantly affected the relationship between expected pain unpleasantness and both concurrent and remembered pain unpleasantness (see interaction term, p ≤ 0.002, Table 2B). A similar trend was found for pain intensity (interaction term: p < 0.06). The subsequent conditional analysis confirmed that expectations significantly predicted placebo analgesia in the REMSD group. We then tested the parametric moderating effect of REM duration in Controls alone, in which there was no floor effect on REM duration. Although this analysis was performed on a small sample, trends were in the same direction, with REM sleep duration affecting the relationship between expected and concurrent (p = 0.13, Table 2C) and remembered pain intensity (p = 0.052, Table 2C). Similar effects were found on concurrent and remembered pain unpleasantness (p = 0.04 and p = 0.06, respectively, Table 2C). The conditional effects confirmed that expectations predicted placebo analgesia at lower REM durations (p ≤ 0.051), whereas this relationship was absent at higher REM durations (p ≥ 0.29).

Taken together, these results support a moderation model, where shorter REM duration leads to stronger expectation-related placebo analgesia.

### Control parameters: vigilance, questionnaires, and blood pressure

Psychomotor vigilance tasks administered at study beginning and end revealed no significant between-group differences in vigilance (group effect for mean reaction time: F(1,25) = 1.78, p = 0.195; median reaction time: F(1,25) = 1.78, p = 0.195, Table 3). However, vigilance decreased in the morning (mean: F(1,25) = 1.78, p = 0.195; median: F(1,25) = 1.78, p = 0.195), with no interaction (mean: F(1,25) = 1.78, p = 0.195; median: F(1,25) = 1.78, p = 0.195). Moreover, subjective sleepiness assessed by the Karolinska Sleepiness Scale (KSS) and Epworth Score Scale (ESS) were comparable between groups (KSS: mean: F(1,25) = 1.78, p = 0.195; ESS F(1,25) = 1.78, p = 0.195, Table 3), along with decreased vigilance (mean: F(1,25) = 1.78, p = 0.195; median: F(1,25) = 1.78, p = 0.195), with no group interaction (mean: F(1,25) = 1.78, p = 0.195; median: F(1,25) = 1.78, p = 0.195). Furthermore, no significant correlation was found with any measures of placebo analgesia, either overall or within groups.

**Table 3:** Psychomotor vigilance task and questionnaires administered in the evening and morning for both groups (mean ± standard error of the mean). Two-sided ANOVAs were used.

	REMSDs				Controls						
	Evening		Morning		Evening		Morning		p		
	Mean	SEM	Mean	SEM	Mean	SEM	Mean	SEM	Group	Time	Interac.
Reaction time: median (ms)	239.86	4.44	283.14	14.3	255.42	6.94	298.12	25.68	0.195	**0.009**	0.564
Reaction time: mean (ms)	251.95	3.98	281.04	11.72	274.95	8.16	329.18	36.70	0.113	**0.025**	0.490
Epworth (/24)	2.31	0.31	3.39	0.29	2.46	0.31	3.46	2.26	0.812	**0.003**	0.903
Karolinska (/9)	3.69	0.47	5.62	0.45	4.08	0.54	5.46	0.69	0.868	**< 0.001**	0.442

All subjects were monitored for diastolic arterial pressure (DAP) and systolic arterial pressure (SAP) throughout the study. As expected, SAP was lower in the morning than evening (F(1,25) = 20.81, p < 0.001, Table 4), but was also lower in REMSDs than in Controls, especially immediately after awakening (F(1,25) = 0.65, p = 0.431; interaction F(1,25) = 4.82, p = 0.014). Thus, the effect was more pronounced at awakening (first morning measurement) than 2 hours later (second morning measurement). Furthermore, no relationship was found between SAP and expected, concurrent, or remembered pain intensity and unpleasantness analgesia. No difference was found for DAP (Time: F(1,25) = 0.59, p = 0.561, Group: F(1,25) = 3.57, p = 0.073, p = 0.431; interaction F(1,25) = 1.20, p = 0.313), or between DAP and expected, concurrent, or remembered pain intensity and unpleasantness analgesia.

**Table 4:** Blood pressure measurements in the evening and morning for both groups (SBP: systolic blood pressure, DBP: diastolic blood pressure, mean ± standard error of the mean). Two-sided ANOVAs were used.

	REMSDs						Controls								
	Evening		Morning 1		Morning 2		Evening		Morning 1		Morning 2		p		
	Mean	SEM	Mean	SEM	Mean	SEM	Mean	SEM	Mean	SEM	Mean	SEM	Group	Time	Interac.
SBP (mmHg)	121.0	2.5	107.3	3.3	111.9	3.2	116.9	2.2	113.2	3.0	114.2	2.5	0.431	**< 0.001**	**0.014**
DBP (mmHg)	64.5	1.8	61.5	2.5	60.0	1.8	65.2	1.6	67.1	1.8	65.6	1.9	**0.073**	0.561	0.313

These results support that neither vigilance nor BP had a significant mediating effect on placebo susceptibility, nor could they explain group differences.

## Discussion

We applied a placebo analgesia induction protocol previously used by us [18] and others [7,32–34] to investigate the effect of selective REM sleep deprivation on the development of placebo responses [21–23]. Results confirm the previously proposed complex and close relationship between sleep, expectations, and placebo analgesia. More specifically, we observed that placebo analgesia improved in the morning in response to REM sleep deprivation, and REM sleep moderated the relationship between pain relief expectations and placebo analgesia.

Laverdure-Dupont and collaborators [18] demonstrated that when volunteers developed positive expectations due to persuasive conditioning and verbal suggestions before sleep, they spent less time in REM sleep, and they showed a high *negative* correlation between REM sleep duration and expectation-mediated placebo analgesia the next day (i.e., less REM sleep associated with strong expectation-related analgesia). However, results were less compelling for volunteers who developed positive expectations due to conditioning and verbal suggestions and who were disturbed by incongruent sensory challenges before sleep. In this group, subjects still expected relief due to the cream, but REM sleep lasted longer, and expectations no longer predicted the magnitude of the placebo response in the morning. Given the proposed role of REM sleep in implicit information reprocessing [35,36], less REM sleep could reflect less need to reappraise information. In light of the results of our previous study, if a new experience is consistent with previously consolidated beliefs, it could be easier to integrate it into a memory network, with reduced requirement for cognitive reappraisal during REM sleep. Based on this previous work, we speculated that REMSD would prevent reprocessing of implicit information obtained from placebo conditioning and verbal suggestions, thus mitigating the need for individual appraisal of information and personal beliefs. We therefore speculated that REMSD enhances both relief expectations and the placebo effect following placebo conditioning and verbal suggestions. In the present study, both the placebo analgesia response and the strength of the relationship between expectations and placebo response were higher following REMSD, but not pain relief expectations. Because they did not have to reappraise implicit information from placebo conditioning and verbal suggestions to align with their previous beliefs and representations, subjects matched their initial relief expectations with their actual perceptions in the morning during placebo testing. In contrast, the control group, who experienced normal REM sleep duration, was able to reappraise the new information.

Concurring with our findings, a recent study revealed alterations in prediction error processing of fear recall following REMSD [37]. More broadly, it is well known that cognitive functioning declines without satisfactory sleep. Prefrontal cortex functions are among the most susceptible [38]. Therefore, in accordance with well-known alterations in frontal executive function following sleep deprivation, including verbal fluency [39], planning [40], logical reasoning, and working memory [38], increased placebo analgesia response could be related to these changes in prefrontal executive functions after REMSD.

However, other mechanisms should be addressed. Prediction error processing is known to be related to learning and motivational behavior [41], which are proposed to be involved in placebo and pain perception [6], suggesting that this complex interrelationship may be a relevant underlying mechanism of the placebo analgesic response following REMSD, as observed in our study. In a brain imaging study, Scott and collaborators [10] tested the correlation between placebo responsiveness and monetary reward and found that placebo responsiveness was related to dopamine activation in the nucleus accumbens. Subjects were then tested for monetary responses in the nucleus accumbens, revealing positive correlations between placebo and monetary responses. Similarly, a recent study showed correlations between ventromedial prefrontal cortex activity during fear conditioning and during REM sleep in healthy volunteers [42], and that ventromedial prefrontal cortex activity during REM sleep mediates the relationship between fear conditioning and extinction the next day. Furthermore, the motivational/reward system is activated during sleep, especially REM sleep, whereby the reward-related mesolimbic regions are activated, including the ventral tegmental area, nucleus accumbens, and anterior cingulate cortex [29]. Sleep deprivation leads to reward and motivation system dysfunction, resulting in significant alternations in behavioral and emotional regulation. Sleep deprivation increased the risk of substance abuse [43] and appetitive behavior [44, 45]. Human studies have reported that insufficient sleep is associated with changes in reward-related decision making: people take greater risks [46], are less concerned with negative consequences of risky decisions [47], and overestimate positive emotional experiences [12]. Collectively, these data suggest that less REMSD may impact neural reward systems so as to exacerbate behavioral reactivity.

In our study, we speculated that REM sleep suppression would alter executive prefrontal functions and/or motivational/reward system reactivity the next morning. However, these mechanisms are not specific to REM sleep [17]. The mechanisms underlying this improved expectation-related placebo response by selective REM sleep deprivation need to be determined in future studies.

The main limitation of this study is that, unlike Laverdure-Dupont and collaborators’ [18] finding that expectations were reinforced following a period of sleep, placebo analgesia in our controls as well as the correlations between their relief expectations and placebo analgesia were low. One possible explanation is that our protocol could have produced an interaction between expectations related to the pain conditioning protocol, with expectations for a medication before bedtime (subjects took either an active or control pill, and were subsequently tested, in addition to receiving an active or control cream on the arm). Although the participants knew little about the effects of the treatment taken before bedtime, they could have developed expectations based on individual experience, possibly resulting in an interaction with the placebo experience. However, this effect does not limit the relevance of our findings nor invalidate the results, because subjects in both groups were probably affected similarly. Another limitation concerns the pharmacological REMSD model based on clonidine absorption [21–23]. Clonidine is known to decrease BP [48] and pain sensitivity and to alter vigilance [49] during wakefulness. However, the effect on pain sensitivity and vigilance appears to be limited to 4 hours post-administration [50]. In our study, although diastolic BP was lower at awakening with clonidine use, the effect was moderate, well within normal range, and had no consequences (i.e., no discomfort following clonidine administration). Moreover, we noted no between-group differences (clonidine or placebo) in morning self-reports of sleep quality, vigilance state, or pain sensitivity at control sites. REMSD did not produce significant changes in total sleep time, slow-wave sleep duration, or sleep fragmentation. This is a strength of the REMSD model, because sleep disruptions (total and partial sleep restrictions, awakening during sleep) may alter pain sensitivity the next day [8], which is particularly relevant because clonidine administration for REMSD interrupts sleep continuity less than the usual sound awakening methods, including touching subjects and calling their name by microphone [20]. Finally, because the NREM sleep deprivation condition was not included in this study, further studies should investigate whether the observed changes in placebo analgesia or its mechanisms are specific to REM sleep.

These findings showed increased placebo analgesia response as well as increased in the strength of the relationship between expectations and placebo response following REMSD. To improve the clinical benefits of placebo analgesia, the contributing factors should be identified and optimized in clinical practice in order to enhance the overall effectiveness of pain treatments. Selective REM sleep deprivation appears to be a promising research avenue. Further studies are needed to better understand how REM deprivation improves the placebo analgesic response after one night, and whether the effect persists.

## Supporting Information

S1 Dataset Psychophysical and polysomnographic data. (XLSX)
